# Membrane microdomains: from seeing to understanding

**DOI:** 10.3389/fpls.2014.00018

**Published:** 2014-02-18

**Authors:** Binh-An Truong-Quang, Pierre-François Lenne

**Affiliations:** Developmental Biology Institute of Marseilles, UMR 7288 CNRS, Aix-Marseille UniversitéMarseille, France

**Keywords:** suppersolution, plasma membrane microdomains, quantitative imaging, multiscale organization, protein clusters

## Abstract

The plasma membrane is a composite material, which forms a semi-permeable barrier and an interface for communication between the intracellular and extracellular environments. While the existence of membrane microdomains with nanoscale organization has been proved by the application of numerous biochemical and physical methods, direct observation of these heterogeneities using optical microscopy has remained challenging for decades, partly due to the optical diffraction limit, which restricts the resolution to ~200 nm. During the past years, new optical methods which circumvent this fundamental limit have emerged. Not only do these techniques allow direct visualization, but also quantitative characterization of nanoscopic structures. We discuss how these emerging optical methods have refined our knowledge of membrane microdomains and how they may shed light on the basic principles of the mesoscopic membrane organization.

## INTRODUCTION

Cell functions strongly rely on its capacity to interact with neighboring cells and extracellular environment. Vital interactions such as metabolic material exchange and biochemical signaling are mediated by the plasma membrane, a semi-permeable barrier which covers the cell surface and separates a cell from its surrounding environment. While the seminal observations of a cell membrane dates back to the early 17th century with the first optical microscopy images of fly eyes and cork tissues (Hooke, 1665), the complexity of the cell membrane is currently being deciphered. The landmark model of the plasma membrane, called the “mosaic fluid model” (Singer and Nicolson, 1972) hypothesizes that plasma membrane is composed of a lipid bilayer housing different freely diffusing membrane proteins. This model implies a homogenous organization of membrane materials, yet did not represent the complete picture. In fact, the plasma membrane is highly asymmetric, with differences in lipid and protein compositions between the inner and the outer leaflet of the bilayer (Rothman and Lenard, 1977; Daleke, 2003). Moreover, the plasma membrane is laterally compartmentalized. Different lipid components can segregate into functionalized microdomains, often referred as lipid rafts (Simons and Ikonen, 1997). Lipid rafts were initially defined as liquid ordered domains enriched in sphingolipid and sterol, and surrounded by a liquid disordered phase (Simons and Ikonen, 1997; Simons and Van Meer, 1988). They can selectively recruit different membrane proteins, such as the glycosyl phosphoinositol (GPI)-anchored proteins (Simons and Van Meer, 1988; Simons and Ikonen, 1997), viral glyco-protein (e.g., Haemagglutinin and neuraminidase; Zhang et al., 2000), thus form protein–lipid microdomains. As cell protein concentration is high [e.g., 23% of the cell surface for red blood cell (Dupuy and Engelman, 2008)], protein–protein interactions can also yield the formation of microdomains, often referred as clusters [e.g., activated Ras-kinases (Prior et al., 2003), immune protein CD2, LAT (Douglass and Vale, 2005; Lin and Shaw, 2005)]. Transmembrane protein clusters can interact with intracellular cytoskeleton network, which can transiently trap proteins during diffusion and act as a “membrane-skeleton fence” (Kusumi et al., 1993, 2005; Sako and Kusumi, 1994). Thus, microdomains and clusters are shared features for lipids and proteins in membranes and are often considered as a “membrane-organizing principle” (Lingwood and Simons, 2010; Mongrand et al., 2010).

Membrane microdomains have been proposed to be essential for different cellular functions. Lipid rafts are proposed to facilitate the apical sorting of different membrane proteins in polarized cell (e.g., epithelial cells). The transport of vesicles enriched with raft markers such as cholesterol and sphingolipids has been shown to be polarized toward the apical surface (Simons and Van Meer, 1988). While some apical proteins have been proven to be preferentially associated with lipid rafts, basal proteins are not. It was suggested that lipid rafts could selectively recruit apical proteins and function as determinant apical landmarks for protein transport during biosynthesis (Simons and Ikonen, 1997). Furthermore, microdomains are important for biochemical reactions of membrane proteins. Compartmentalization by microdomains and clusters may provide a local optimal environment to facilitate the speed and efficiency of these reactions (Stier and Sackmann, 1973; Klausner et al., 1980; Karnovsky et al., 1982; Simons and Sampaio, 2011). Also, confinement by microdomains would allow receptors and cofactors to meet faster and therefore speed up cell responses (Batada et al., 2004). Clustering of receptor tyrosine kinases (RTKs) upon ligand binding has been shown to be essential for the activation of kinases, which promote downstream signaling cascades (Saha et al., 2007). Ephrin receptors form the largest subfamily of RTKs regulating cell shape, movement and attachment. Upon binding to Ephrin ligands, Ephrin receptors accumulate into highly packed microdomains, which generate clearly defined signaling centers at cell–cell interfaces (Saha et al., 2007; Seiradake et al., 2010). Perturbation of Ephrin receptor clustering by point mutation in the binding interfaces of extracellular domains results in homogenous cell surface distribution with a loss of clusters at cell–cell contacts and yields disruption of signaling cascades (Seiradake et al., 2010). Another important member of RTKs is the epidermal growth factor receptor (EGFR), which is implicated in cell growth, proliferation, and differentiation to cell survival (Ullrich and Schlessinger, 1990; Yarden and Sliwkowski, 2001; Hoeller et al., 2005). Binding of EGF to its receptor EGFR leads to receptor dimerization, followed by tyrosine phosphorylations of the receptor (Pawson, 2004) and assembly of the protein complexes which activate intracellular signaling (Blagoev et al., 2003; Jones et al., 2006). In EGFR clusters, the number of phosphorylated EGFRs become larger than the number of EGF ligands, as unliganded EGFRs are also phosphorylated, implying an amplification of EGF signaling (Ichinose et al., 2004). Microdomains also serve as platforms for receptor internalization, thus modulate the sensitivity of cell signaling or the affinity of cell–cell adhesion during tissue morphogenesis (Klaasse et al., 2008; Levayer et al., 2011). In addition, a micro-scale organization is hypothesized to be the entry port for viruses (Mañes et al., 2003) and plays important roles in immunological response (Dykstra et al., 2003). In calcium signaling, formation of Ryanodine receptor (RyR) clusters are required for Ca^2^^+^ sparks (Cannell et al., 1995), which is required for muscle contraction and neurotransmission (Baddeley et al., 2009). In the context of cell adhesion, microdomains, or clusters of adhesion molecules are essential for supporting tensile forces during cell migration (Maheshwari et al., 2000; Roca-Cusachs et al., 2009) and cell shape changes (Cavey et al., 2008; Rauzi et al., 2010). Together, these reports showed that the spatial organization of membrane proteins into microdomains can play crucial roles in a large range of biological processes.

Over the last few decades, a large number of studies on membrane models and extracted cell membranes have led to the hypothesis that cell membranes are heterogeneous and microstructured. The co-existence of liquid-ordered and liquid-disordered phases has been first documented on membrane models and extracted cell membranes by using different physical methods including electron spin resonance (ESR; Stier and Sackmann, 1973; Marsh et al., 1982; Ge et al., 2003; Swamy et al., 2006), different scanning calorimetry (DSC; Mabrey and Sturtevant, 1976; Melchior, 1986; Wolf et al., 1990), X-ray (Wunderlich et al., 1978; Gandhavadi et al., 2002), nuclear magnetic resonance (NMR; Mitrakos and MacDonald, 1996, 1997; Veatch et al., 2004) electron microscopy (Hui and Parsons, 1975; Hartmann et al., 1977; Prior, 2003). Biochemical methods such as detergent-soluble membranes and crosslinking assays (Brown and Rose, 1992; Cerneus et al., 1993) have been extensively used and often been over-interpreted as a criterion for the existence of lipid microdomains in cell membranes. *In situ* measurements using fluorescence microscopy methods, such as fluorescent polarization or fluorescent life time imaging microscopy (FLIM) used to study fluorescent lipid analogs have shown the co-existence of different lipid phases (Fiorini et al., 1988) and their organization in sub-resolution domains in the plasma membrane (Owen et al., 2012). In addition, the dynamics of membrane proteins revealed by fluorescence recovery after photobleaching (FRAP; Wolf et al., 1981; Metcalf et al., 1986), fluorescent correlation spectroscopy (FCS; Fahey et al., 1977; Meyer and Schindler, 1988; Korlach et al., 1999; Schwille et al., 1999), and single particle tracking (SPT) using optical (Kusumi et al., 1993) or fluorescent labels (Schutz et al., 2000; Jaqaman et al., 2011; Suzuki et al., 2012) have demonstrated multiple modes of diffusion: different diffusion coefficients or different types of motion (i.e., confined/Brownian) for a single protein species (Metcalf et al., 1986; Schwille et al., 1999) or for lipid analogs (e.g., saturated and unsaturated lipid probes) which partition in different lipid phases (Wolf et al., 1981; Schutz et al., 2000). These observations prime the hypothesis that there are local heterogeneities, such as “pinball in pinball machine” (Jacobson et al., 1995; Sheets, 1995) with microdomain obstacles or “membrane-skeleton fences” (Kusumi et al., 1993; Sako and Kusumi, 1994) mediated by protein-cytoskeleton interactions. Moreover, at the molecular scale (i.e., 2–10 nm), Förster resonance energy transfer (FRET) experiments have supported the existence of small tightly packed clusters of membrane anchored and transmembrane proteins with size of few 10s nanometers (e.g., <70 nm in case of GPI-anchored proteins) containing only few proteins (Damjanovich et al., 1997; Varma and Mayor, 1998; Sharma et al., 2004; Gowrishankar et al., 2012).

While current data strongly support the existence of microdomains/clusters of different kinds, some of the methods cited above are prone to artifacts or have various limitations when used to characterize microdomains. First, concerning the spectra-based methods (e.g., ESR, DSC, X-ray, NMR), the calibrated spectra obtained from very simple membrane models composed of only a few types of lipids at predefined ratio are often too simplistic to interpret the spectra obtained on cell membranes, which are far more complex in terms of lipid and protein compositions. Second, biochemical methods, such as nonionic detergent-soluble assays, can induce artificial clustering (Heerklotz, 2002). Third, although capable of providing nanometric resolution, electron microscopy suffers from low specificity and artifacts caused occasionally by long and invasive sample preparation. Fourth, the interpretation of FRAP and FCS data are generally model-dependent. Fifth, SPT cannot always distinguish between alternative models of membrane organization, if they show similar single molecule dynamics. Finally, while the conventional fluorescence imaging methods such as confocal microscopy provide direct imaging of membranes *in vivo*, they fail to resolve domains of nanometric sizes and cannot be used to assess the models inferred from FRET or FCS measurements. This failure arises from the fundamental limit of diffraction, which sets a criterion of the minimum resolvable distance between two punctual objects (Abbe, 1873; Rayleigh, 1874). Molecules closer than this limit, ~200–350 nm (for optical wavelengths), cannot be distinguished. Thus, new methods that manage to circumvent this optical limit are required for direct visualization and quantification of nanoscopic organization of membrane domains. Fortunately, during the last few years, different strategies have been proposed and have successfully improved the spatial resolution to one tenth of the diffraction limit. In the following sections, we will review these so-called “superresolution” optical methods and discuss how they contribute to our understanding of the mesoscopic organization of the plasma membrane. While, little has been done yet with these new approaches on the membranes of plant cells, recent works on animal cells that we present here will hopefully pave the way for the plant community.

## SUPERRESOLUTION USING SPATIAL MODULATION

**FIGURE 1:**
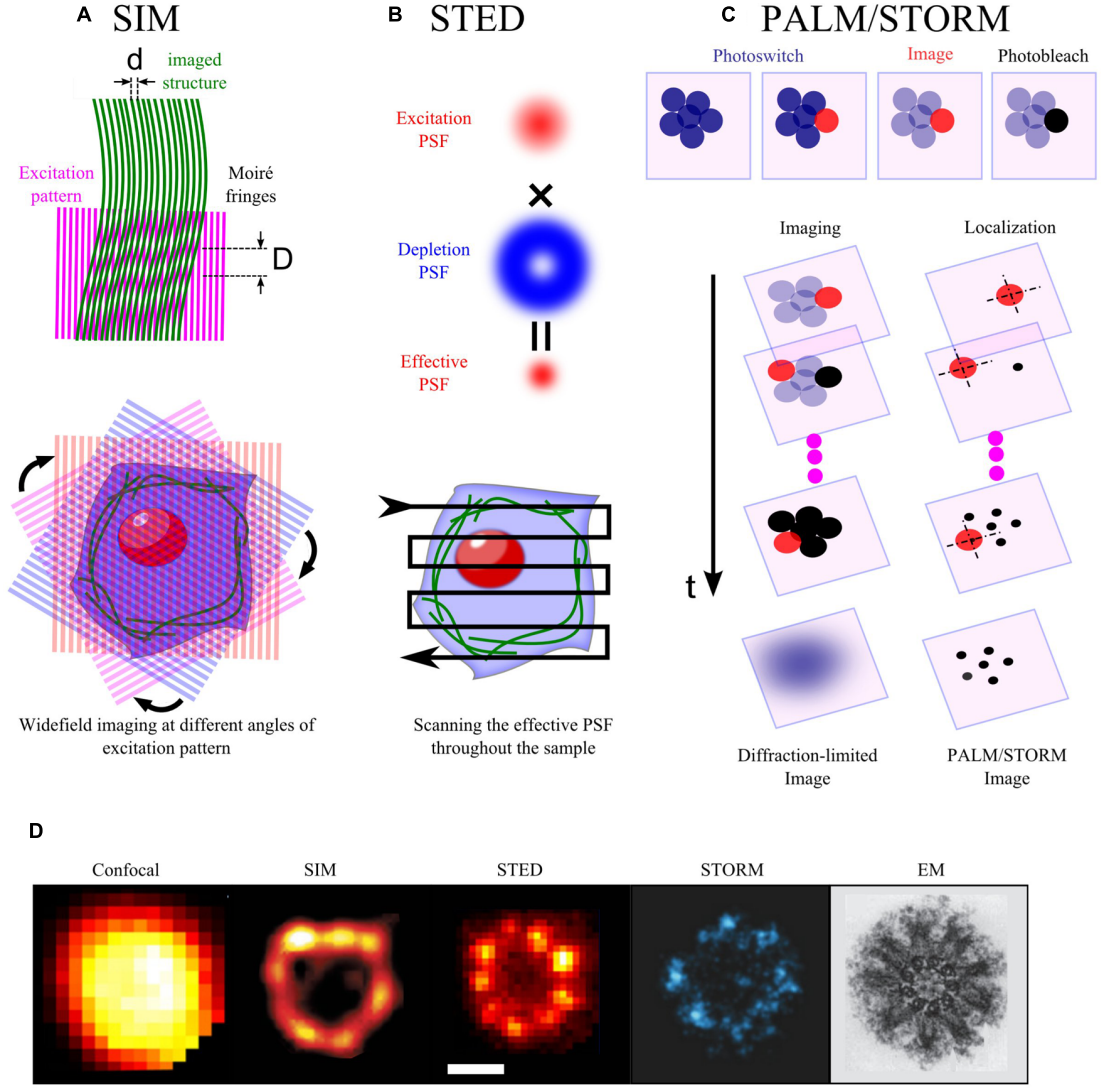
**Basic principles of some superresolution optical methods. (A)** When two line patterns are superposed multiplicatively, a beat pattern or Moiré fringes are formed with smaller spatial frequency than the initial patterns (upper panel, *d* < D). In SIM, the sample plane is excited by a patterned wide-field illumination (commonly in striped-shaped pattern). This pattern combines with the sample structural pattern to form Moiré fringes. Thus, high spatial frequency information of the sample, which is below the diffraction limit, is decoded into a lower frequency Moiré pattern. Moiré patterns obtained by rotating the excitation pattern at different angles are collected and used for a computational reconstruction of a higher resolution images (lower panel). **(B)** In STED, the sample plane is illuminated simultaneously by an excitation and a depletion beam. The depletion beam in a donut-shape allows to deplete non-linearly most of the excited molecules into the dark state through stimulated emission, but leaves intact the center of the excitation PSF. This thus yields an effective PSF, which is smaller than the initial diffraction-limited PSF. The sample is scanned with this effective PSF to form a superresolution image. **(C)** Single molecules in a crowded biological sample can be isolated by photoswitching (from dark to bright or from one color into another). The photoswitched molecules are then imaged and localized before being photobleached (upper panel). The procedure is iteratively repeated until all of the initial molecules are imaged and photobleached. The superresolution image is reconstructed from the position list of all localized molecules (lower panel).** (D)** Images of the centriole taken with different methods: confocal and STED images (Lau et al., 2012), SIM image (Lawo et al., 2012), STORM image (Mennella et al., 2012), EM image (Hagan and Palazzo, 2006).****

Improvement of the resolution can be obtained by spatial modulation of the excitation light. By exciting the sample plane with a series of patterns, structured illumination microscopy (SIM) can decode the conventional inaccessible high-resolution structural information into Moiré images obtained by individual excitation pattern (**Figures 1A,D**) and then allow the reconstruction of images at higher resolution (Gustafsson, 2000). This method allows to achieve a twofold increase in resolution in 2D (Gustafsson, 2000) or 3D (Gustafsson et al., 2008) with linear excitation, and even a theoretical unlimited increase in resolution in the nonlinear excitation regime (Gustafsson, 2005; Rego et al., 2012). SIM has been used to visualize the punctate organization of antigen membrane glycoprotein (Hammonds et al., 2012), lipid rafts (Svistounov et al., 2012) of 100 nm in size and structure of nanopores in plant cells (Fitzgibbon et al., 2010). Yet, there are two main challenges for SIM. First, to improve the resolution, multiple (10 ~ 100) excitation patterns are required per imaging plane, thereby fundamentally limiting the acquisition rate. Second, the reconstruction of superresolution image requires complicated and time consuming computational post-analysis, in particular for 3D image. Note that recent analog implementation using micro-array lenses for on-line optical analysis can eliminate the need to acquire and digitally combine multiple camera exposures, thereby improving time-resolution down to few 10s milliseconds (York et al., 2013).

Resolution can also be increased in another elegant way. In fluorescence microscopy, the size of the focal spot at the sample plane usually defines an unresolvable region. The resolution can, however, be improved by reducing the size of the region from which the excited molecules fluoresce. By superimposing a hollow-patterned laser (e.g., donut-shape) on the conventional excitation laser to specifically quench excited molecules at the rim of the focal spot through stimulated emission (**Figures 1B,D**), stimulated emission depletion (STED) microscopy can improve the lateral resolution down to a few ten nanometers (Hell and Wichmann, 1994; Klar et al., 2000; Westphal and Hell, 2005). Furthermore, the same principles can be applied to improve the axial resolution with a depletion phase mask acting along the optical axis. Combination of lateral and axial depletion beams allows to obtain isotropic resolution (3D-STED, Harke et al., 2008; Hein et al., 2008; Wildanger et al., 2009).****2D-STED microscopy has revealed a large set of synaptic or membrane-bounded protein microdomains of 50–60 nm in size (Sieber et al., 2006, 2007; Willig et al., 2006; Demir et al., 2013). Furthermore, by implementing FCS in variable STED nanometric observation volumes, as done previously with diffraction-limited spots (Wawrezinieck et al., 2005; Lenne et al., 2006), STED microscopy has further characterized the size (<20 nm) and lifetime of lipid rafts (~10 ms; Eggeling et al., 2009). While STED can practically increase resolution by five fold as compared to classical confocal microscopy, it is limited in the speed of acquisition (~0.1–1 Hz). High laser power of the depletion laser (10^4^–10^7^W/cm^2^ for pulse peak intensity) can be very toxic for live samples and can cause photo-bleaching during imaging. Finally, the efficiency of STED effect requires a perfect alignment of the excitation and the depletion lasers, which might be complex to achieve.

## SUPERRESOLUTION USING TEMPORAL MODULATION

Spatial resolution can alternatively be improved by modulating/switching the emission of fluorescent molecules. The rationale of this approach is that the position of a single fluorescent molecule can be determined with a precision much better than the resolution criterion imposed by the diffraction limit, if the number of collected photons per molecule is high (Thompson et al., 2002). In a dense material, simultaneously excited molecules separated by distances smaller than the diffraction limit cannot be individually localized due to the spatial overlap of their fluorescence signal. If only a sparse subset of fluorescent molecules, separated by distances larger than the diffraction limit is activated at one time of acquisition, they can be localized individually with high precision. The whole population of fluorescent molecules can thus be localized by successive acquisitions, using temporal modulation/switching of fluorescence emission, thereby providing a map of single molecules and an image at super-resolution (**Figures 1C,D**). To date, there are two types of microscopies, which implement these principles using photoswitchable molecules. The first type called photoactivated-localization microscopy (PALM), is based on photoactivable fluorescent proteins (PAFPs; Betzig et al., 2006). Upon irradiation by appropriate activation laser (e.g., UV laser), PAFPs can shift their spectral emission from one color to another [e.g., EosFP (Wiedenmann et al., 2004), Dendra (Gurskaya et al., 2006)] or from dark to bright states [e.g., Dronpa (Ando et al., 2004), PamCherry (Subach et al., 2009)]. The second type called stochastic optical reconstruction microscopy (STORM; Rust et al., 2006), is based on photo-switchable organic probes: fluorescent activator/reporter probe (e.g., cy3/cy5) can undergo multiple fluorescent cycles between dark and bright states triggered by excitation and activation lasers (e.g., 657/532; Rust et al., 2006). In a variant form of STORM called “direct” STORM (dSTORM), conventional fluorophores can also be “directly” reversibly recycled between fluorescent and dark states by irradiation with a single wavelength and the use of a reducing buffer without any need of activator fluorophore (Heilemann et al., 2008). The axial resolution along the optical axis can also be greatly improved up to a few 10 nm using astigmatic detection (Huang et al., 2008), bi-plane (Juette et al., 2008), or more sophisticated interfometric methods (Shtengel et al., 2009; Kanchanawong et al., 2010). In live mode for slowly moving structures, PALM/STORM has provided kinetic data on nanoclusters with a spatial resolution of 60 nm and a time resolution down to 25 s (Shroff et al., 2008). Alternatively, by tracking photoactivated molecules, single particle tracking-PALM (sptPALM) can obtain few orders of magnitude more trajectories per cell in comparison with traditional SPT, therefore can create a map of the dynamic heterogeneity in cell membrane (Manley et al., 2008). Furthermore, if molecules are photoactivable only once, PALM provides quantitative counting of single molecules, thereby allowing the measurement of the density or even the stoichiometry of microdomains’ components.

Although so far, localization-based microscopy (PALM/STORM) has provided the best spatial resolution among other super-resolution optical microscopy techniques, its time resolution is still limited (>0.05 Hz) due to the need to collect a large amount of single molecule images. Furthermore, PALM/STORM data require cautious interpretation. There are indeed concerns about clustering artifacts, which might arise from the oligomeric nature of fluorescent tags (McKinney et al., 2009; Zhang et al., 2012). Moreover, some fixation methods fail to immobilize lipids and to a less extent lipid anchored proteins and some signaling proteins (Tanaka et al., 2010). Clustering could then arise from artificial antibody-induced processes. In addition, fluorescent molecules can exhibit photoblinking, which results in artificial clustering due to multiple detection of the same molecule (Annibale et al., 2011). Finally, localization of single molecules in a high background noise, correction for sample drift during long acquisition periods (10–30 min) and data visualization/analysis from the list of localized proteins are often complicated and time consuming.

The development of localization microscopy (PALM and STORM) is currently a very active field with rapid development in different areas. The possibility to photoswitch a large range of commercially available fluorescent tags using reducing buffer without the need of oxygen scavengers (Heilemann et al., 2009; van de Linde et al., 2011) makes it an extremely convenient method for multicolor superresolution imaging. In addition, coupling of PALM and dSTORM provides simultaneous access to molecular counting of PALM and higher resolution imaging of dSTORM which use brighter and less photobleaching organic fluorophores compared to fluorescent proteins (Izeddin et al., 2011; Muranyi et al., 2013). Brighter versions of fluorescent proteins [mEos3 (Zhang et al., 2012), PSmOrange2 (Subach et al., 2012), mGeos-M (Chang et al., 2012)] and fluorophores (Lukinavičius et al., 2013), optimization of imaging buffer [e.g., Heavy water (Lee et al., 2013), cyclooctatetraene (Olivier et al., 2013a), Vectashield (Olivier et al., 2013b)], and labeling strategy [e.g., Nanobody (Ries et al., 2012)], have pushed further the spatial and temporal resolution limits.

In parallel with the improvement of imaging techniques, new algorithms for image analysis have been proposed. Real-time analysis requires the implementation of new algorithms for fast detection and localization of single molecules from large series of images. Localization is speed-up by replacing the classical Gaussian kernel-based fitting algorithm by the classical Högbom “CLEAN” algorithm in QuickPALM (Henriques et al., 2010), the fluoroBancroft algorithm in livePALM (Hedde et al., 2009), radial symmetry center (Parthasarathy, 2012), or wavelet segmentation (Kechkar et al., 2013) and also by implementation of parallel computational structures such as graphical processing unit (Smith et al., 2010). In addition, the precision of single molecule localization in a highly dense sample, in particular for STORM, has been significantly increased. Single molecule positions are retrieved by fitting overlapped spots with a multiple PSF, either using a maximum likelihood estimation in DAOSTORM (Holden et al., 2011), Bayesian statistics (Cox et al., 2012) or a global optimization using compressed sensing, which does not require any assumption on the number of molecules in the image (Zhu et al., 2012). Alternatively, the superresolution image can be obtained by iterative image deconvolution in place of single or multiple emitter localization (Mukamel et al., 2012). Furthermore, new toolboxes for analyzing complex patterns of protein organization using pair-correlation analysis (e.g., PC-PALM, Sengupta et al., 2011, 2013; Veatch et al., 2012) or for visualization of 3D PALM/STORM data using surface rendering (Beheiry and Dahan, 2013) are now available to the scientific community. The use of monomeric fluorescent tags (Zhang et al., 2012), monovalent antibodies or purified Fab fragments (Chojnacki et al., 2012), and new computational algorithm for photoblinking correction (Annibale et al., 2011; Lee et al., 2013) have significantly reduced clustering artifacts.****In the near future, the combination of PALM/STORM with EM (Watanabe et al., 2011) or FRET (Renz et al., 2012) will be very useful to characterize the supramolecular organization membrane microdomains.

## NANOSCOPIC ORGANIZATION: A SHARED FEATURE BY LIPIDS AND PROTEINS

During the past decades, the study of membrane organization has been mainly focused on lipid organization, the putative lipid rafts being emphasized as a “stereotype” of membrane domains. Focus on lipid rafts has masked to some extent the existence and the role of protein clustering in membranes. In addition, the lack of direct visualization evidence together with the recognition of possible experimental artifacts has raised doubts about the existence of microdomains/clusters.

**FIGURE 2:**
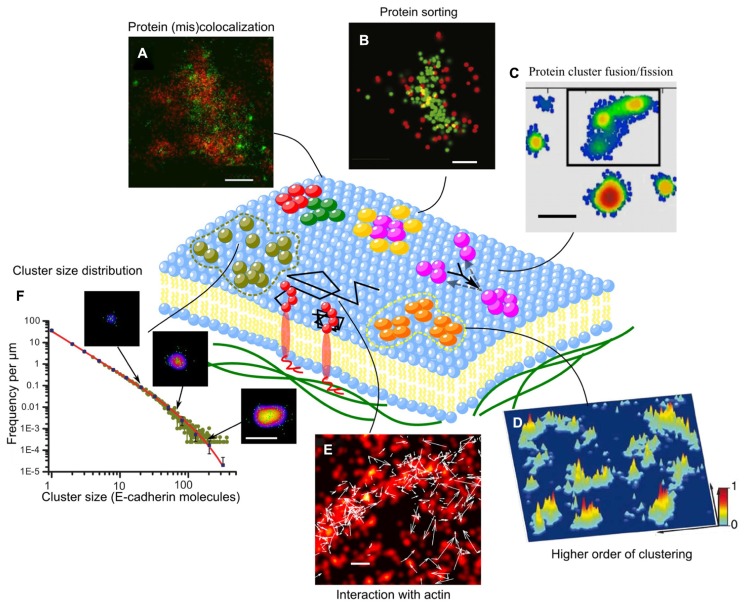
**Supramolecular organization and dynamics of membrane proteins. (A)** α-actinin and vinculin only partially colocalize within each focal adhesion. While α**-**actinin exists in large patches emanating from stress fibers, vinculin coalesces in small, dense clusters scattered across each focal adhesion (Shroff et al., 2007). **(B)** Images of individual LAT clusters showing preferential organization of SLP-76 at the rims of LAT clusters (Sherman et al., 2011); **(C)** Plot of a representative membrane showing the distribution and inner gradients of several syntaxin clusters. Black frame: a super-cluster composed of three smaller clusters that might be in the process of uniting (Bar-On et al., 2012); **(D)** Three-dimensional probability density plots for CD3ϖ-PSCFP2 in native plasma membrane sheets from activated T cells on immobilized surfaces. Molecules are presented as a normalized Gaussian probability density distribution with a width equal to their positional accuracy. Height and color represent the probability density at that point (*x*, *y*), with the highest probability density of all images set as 1 (Lillemeier et al., 2010); **(E)** Superimposed two-color (Dendra2-HA and PAmCherry-actin) live cell FPALM image of PAmCherry-actin (red pseudocolor) and trajectories of Dendra2-HA molecules (white arrows) in a living NIH3T3-HAb2 fibroblast at 37°C (Gudheti et al., 2013); **(F)** Cluster size distribution of E-cadherin clusters and the power-law with exponential cut-off fitting (solid line). Sample images of clusters are shown with arrows that indicate cluster size (Truong-Quang et al., 2013). Scale bars, 500 nm **(A)**, 200 nm **(B,C,E,F)**, and 1 μm **(D)**.

Importantly, superresolution optical microscopy has supported the raft-hypothesis by providing direct evidence of lipid rafts *in vivo* as well as characterization of their dynamics when used in combination with other F-approaches such as FCS or FLIM (Eggeling et al., 2009; Mizuno et al., 2011; Owen et al., 2012). Superresolution imaging has also provided evidence of the nanoscopic organization of a large set of membrane proteins, ranging from immune (Lillemeier et al., 2010; Sherman et al., 2011; Scarselli et al., 2012), adhesion (Betzig et al., 2006; Shroff et al., 2007), viral (Manley et al., 2008; Lehmann et al., 2011), synaptic (Willig et al., 2006; Sieber et al., 2007), and chemotaxis (Greenfield et al., 2009) protein clusters (**Figures 2A–D**; **Table 1**). These microdomains/clusters have been observed not only in fixed but also in live cells (**Figure 2E**; Hess et al., 2007; Shroff et al., 2008; Hein et al., 2010) ruling out the possibility of artifacts caused by the fixation procedure (Hess et al., 2005; Lee et al., 2011). Interestingly, some proteins, such as adrenergic receptor β2-AR or viral protein HIV-1, do not colocalize with lipid raft markers suggesting raft-independent clustering (Lehmann et al., 2011; Scarselli et al., 2012). Thus, further application of multicolor superresolution and specific perturbation of lipid/protein to explore the contribution of protein–protein versus protein–lipid interactions to the formation of protein microdomains would be extremely informative.

**Table 1:** Applications of superresolution microscopy to detection and quantification of the supramolecular organization in the plasma membrane.

Measurements	Methods	Spatial resolution	Temporal resolution	Observations and reference
Dimensions	SIM	100–150 nm in *x*–*y*, 200–300 nm in *z*	na	70–150 nm clusters of the antiretroviral membrane protein tetherin (Hammonds etal., 2012); 100 nm lipid raft microdomains (Svistounov etal., 2012). 200–500 nm clusters of membrane-bounded proteins in transgenic tobacco cells (Fitzgibbon etal., 2010).
	STED	50–70 nm	na	70–80 nm clusters of the calcium sensor synaptotagmin (Willig etal., 2006); 50–60 nm clusters of the vesicle docking protein Syntaxin (Sieber etal., 2006,^2007^); 150 nm clusters of the cAMP signaling protein ACIII (Yang etal., 2013); 100 nm nanoclusters of the phos-phatase protein ABI1 and protein kinase CPK21 in *Arabidopsis thaliana *cells (Demir etal., 2013).
	PALM	10–30 nm	na	100–150 nm clusters of Vinculin in focal adhesions (Betzig etal., 2006); 100 nm lipid raft (Mizuno etal., 2011); 100 nm clusters of the G-protein receptor GPCR (Scarselli etal., 2012, 2013); 100 nm clusters of the retroviral protein Gag and antiretroviral protein tetherin (Lehmann etal., 2011); 35–70 nm clusters of the T cell receptor (Lillemeier etal., 2010).
	STORM	20–30 nm	na	65–105 nm clusters of viral envelope protein Env (Roy etal., 2013); <300 nm clusters of immune receptor TLR4 (Aaron etal., 2012).

Multicomponent organization	STED	40 nm	na	Different clustering states of the viral envelope protein Env around the core protein Gag, corresponding to different maturation stages of viral particles (Chojnacki etal., 2012); Synapsin forms cluster inside or outside synaptic vesicles (Kempf etal., 2013).
	PALM	20–30 nm	na	Different levels of colocalization of the cargo protein transferin with the vesicle coat clathrin (Subach etal., 2009); nanoclusters of vinculin, paxillin, zyxin (in focal adhesion) have interwoven arrangements with little overlap (Shroff etal., 2007); the adaptor protein SLP-76 localizes at the periphery of immune protein Lat nanoclusters (Sherman etal., 2011).
	PALM/STORM	15–20 nm	na	The highly adhesive isoform of AQP-4 (aquaporin channel) forms the core of 50–130 nm clusters and is surrounded by a less adhesive isoform (Rossi etal., 2012); 50–150 nm HIV-Gag clusters are surrounded by the viral envelope protein Env (Muranyi etal., 2013).
	iPALM	<20 nm isotropic	na	Focal adhesion complexes have a three-layer structure: a membrane-apposed signaling layer containing integrin, focal adhesion kinase, and paxillin; an intermediate force-transduction layer containing talin and vinculin; and an uppermost actin-regulatory layer containing zyxin, vasodilator-stimulated phosphoprotein and alpha-actinin (Kanchanawong etal., 2010).

Multiscale organization	STED	40 nm	na	40–60 nm adhesion clusters spaced by 100 nm, accumulate inside focal adhesions of few micrometers (Rönnlund etal., 2013).
	PALM	20–30 nm	na	100–200 nm clusters of vinculin, paxillin, zyxin accumulate to form focal adhesions of few micrometers (); the intercellular adhesion protein E-cadherin forms clusters of a few 10s to a few 100s molecules, which accumulate into micrometer adhesion puncta (Truong-Quang etal., 2013). 100 nm clusters of the immune protein TCR, LatA, ZAP-70 in immunological synapses of a few micrometers (Lillemeier etal., 2010; Sherman etal., 2011); nanoscopic clusters of Tar, CheY, CheW accumulate at the two ends of *E. coli* bacteria to form micrometer-scale clusters (Greenfield etal., 2009); The viral protein hemagglutinin forms clusters with size ranging from 40 nm up to a few micrometers (Hess etal., 2007).
	dSTORM	20 nm	na	Syntaxin 1 or SNAP-25 (synaptic proteins) form 90–130 nm clusters, whose molecular density gradually decreases from the core to the periphery. Large-clusters show several density gradients, suggesting that they are formed by fusion of several clusters (Bar-On etal., 2012).

Kinetics of assembly/disassembly	SIM	100 nm	s	Dynamic assembly of the membrane bound DNA translocase SpolllE protein, in *B. subtilis* bacteria (Fiche etal., 2013).
	Live STED	40 nm	10 s	Clusters of the cell membrane proteins caveolin and connexin-43, from 50 to a few hundreds nanometers in size in living cells (Hein etal., 2010).
	FCS-STED	30 nm	<us	Anomalous diffusion of lipid analogs in membrane models or in living cells (Eggeling etal., 2009; Mueller etal., 2011; Leutenegger etal., 2012; Sezgin etal., 2012; Honigmann etal., 2013).
	Live PALM	60 nm	25 s	100–300 nm clusters of the focal adhesion protein Paxillin exhibit growth, fusion, and dissolution on the order of a few minutes to a few 10s of minutes time scale (Shroff etal., 2008).
	sptPALM	20–30 nm	30–100 ms	Dynamic heterogeneity of the viral protein Gag with a mobile fraction and an immobile fraction confined in 100–200 nm clusters (Manley etal., 2008); the trajectories of the cargo proteins TfR or EGFR overlap those of the vesicle coat Clathrin (Subach etal., 2010); Colocalization of the (non-mobile) Hemagglutinin viral protein with actin-rich membrane regions (Gudheti etal., 2013).
	Live STOM	30 nm in *x*–*y*; 50 nm in *z*	0.5–2 s	Dynamic assembly of endocytic vesicles with 70 nm clusters of the cargo protein Transferrin, surrounded by 150 nm clusters of the vesicle coat Clathrin (Jones etal., 2011).

Cluster size (number of molecules) distribution	PALM	20–30 nm	na	Size distribution ranging from a few to a few 10s of proteins of the antiretroviral protein Tetherin (Lehmann etal., 2011); the T cell receptor (Sherman etal., 2011; Lillemeier etal., 2010; a G-protein signaling receptor (Scarselli etal., 2012); exponential distribution of chemotaxis proteins Tar, CheY, CheW (Greenfield etal., 2009); power-law distribution of the cell–cell epithelial adhesion protein E-cadherin (Truong-Quang etal., 2013).
	STED	50 nm	na	Clusters of ~75 syntaxin molecules (Sieber etal., 2007); clusters of ~7–10 of viral envelope protein Env trimers (Chojnacki etal., 2012).

## THE DYNAMICS OF MICRODOMAINS

The possibility to observe nanoscopic domains in live cells has brought unprecedented information on their dynamics. Live-superresolution microscopy has been used to demonstrate a wide range of morphological and dynamic behaviors, which depend on the types of proteins, subcellular environments, and cell types. For example, adhesion complexes (e.g., Paxillin) have been shown to form either elongated structures with size up to few micrometers or point-like puncta of 100–300 nm in size. While elongated nascent adhesion complexes exhibit growth, fusion and dissolution at few minutes time scale, the punctae can be stable during a few 10s of minutes. The dynamics of theses structures differs from one cell type to another (e.g., CHO and 3T3 fibroblast cells) with different protrusive motions (Shroff et al., 2008). Some other types of membrane microdomains have much higher dynamics. The assembly of endocytic cargo proteins (e.g., Transferrin ) is on the time scale of a few 10s of seconds, and the life time is on the time scale of 1 min (Jones et al., 2011). Furthermore, single molecule tracking has revealed heterogeneities in membrane protein dynamics. While non-clustering proteins (e.g., VSVG-protein) exhibits a rather homogenous diffusion map, clustering proteins (e.g., Viral protein Gag) show distinct zones of free diffusion and immobile behavior, suggesting a protein-trapping mechanism by microdomains (Manley et al., 2008). In this way, microdomains of vesicular decorating proteins, such as Clathrin, can recruit specific cargo proteins (e.g., Transferrin receptor TfR and epidermal growth factor receptor EGFR) with specific targeting sequence (Subach et al., 2010). Protein-trapping can be mediated by passive protein–protein interactions inside microdomains, but can also arise from interactions with the underlying active actin cytoskeleton (**Figure 2E**; Gudheti et al., 2013).

## NANOSCOPIC ORGANIZATION OF MULTI-COMPONENT MICRODOMAINS

Superresolution optical microscopy has made possible the simultaneous visualization of different protein organization at the nanoscopic scale (**Table 1**). Measuring the relative distance between protein components in complexes is key to determining the existence of molecular interactions. Some proteins, which colocalize at the diffraction-limited resolution turn out to have little overlap or even to create interwoven arrangements as revealed by high-resolution image (Shroff et al., 2007). Proteins that have similar functions (e.g., vinculin and alpha-actinin) show some degree of nanostructural overlap, while functionally distinct proteins (e.g., Paxilling and actin) exhibit very little overlap (**Figure 2A**). Also, proteins that were suggested to be in separate microdomains as observed by immunoelectron imaging, a method which is prone to clustering artifact (D’Amico and Skarmoutsou, 2008), turn out to have a significant overlap depending on the activating cell state. As proteins interact at short distances, spatial overlap at the nanometer scale is a condition to direct biochemical reactions (Lillemeier et al., 2010; Sherman et al., 2011), which might then initiate massive recruitment, e.g., by docking of synaptic vesicles at the reaction site, and activation of downstream signals (Purbhoo et al., 2010; Williamson et al., 2011). Different levels of colocalization between nanoclusters of proteins, such as vesicular coats and cargo proteins, are indicative of different stages of maturation of the endocytic machinery (Subach et al., 2009). Moroever, multi-protein microdomains can have internal structure: for example the Adaptor protein SLP-76 localizes at the rim of nanoclusters of the immune protein LAT (**Figure 2B**; Sherman et al., 2011), suggesting a protein-sorting mechanism. The peripheral proteins prevent further accumulation of the core proteins and therefore control the growth of microdomains, as being reported for nanoclusters of aquaporin channel (Rossi et al., 2012). Furthermore, protein complexes in a microdomain can be structured along the transverse direction into nanoscopic composite multilaminar protein architecture. For example, focal adhesion complexes are composed by three layers: a membrane-apposed signaling layer containing integrin, focal adhesion kinase and paxillin; an intermediate force-transduction layer with talin and vinculin; and an uppermost actin-regulatory layer with zyxin, vasodilator-stimulated phosphoprotein, and alpha-actinin (Kanchanawong et al., 2010).

## MULTISCALE ORGANIZATION OF MICRODOMAINS

Protein–protein interactions and cooperation depend critically on their relative distance, and therefore on molecular packing in microdomains. In fact, changes in lateral packing of chemoreceptor arrays significantly affect bacterial chemotaxis response (Khursigara et al., 2011). Similarly, modulation of intermolecular distances between the cell-matrix adhesion protein integrin using nanopatterned substrates can amplify or suppress the adhesion force (Selhuber-Unkel et al., 2010). Although, the resolution limit of PALM/STORM (e.g., 20 nm) is larger than the size of most membrane proteins (e.g., ~5–7 nm), molecular packing/density in clusters can still be inferred from the the number of proteins counted in a cluster area. As an example, based on quantitative PALM data, the clusters of the cell–cell adhesion molecule E-cadherin was found to be tightly packed *in vivo* (Truong-Quang et al., 2013)*. *Molecular packing of microdomains can vary dependent on the type of proteins (e.g., Between GPI-anchored protein and signaling protein Lyn, Lat; Sengupta et al., 2011). Density of molecules can be also significantly different in the center and at the periphery of microdomains (Bar-On et al., 2012).

Interestingly, for a large range of proteins, highly packed microdomains do not distribute randomly but tend to form larger clusters, that may explain previous observations of larger microdomains at the microscale [e.g., Immune synapse (Monks et al., 1998; Davis et al., 1999) or adhesion complexes (Zaidel-Bar et al., 2003; McGill et al., 2009)]. For example, nanoclusters of cell-matrix adhesion complexes (e.g., Vinculin, Paxillin, Zyxin) of 100–200 nm in size (Betzig et al., 2006; Shroff et al., 2007) accumulate to form focal adhesion of few micrometers in size. Similarly, immune proteins (e.g., TCR or LatA) form clusters of a few 10s of nanometers, which concentrate, with a proximity of about 100 nm, in the micrometer-scale immune synapse (**Figure 2D**; Lillemeier et al., 2010). The same observation has been made for the viral protein Hemagglutinin (Hess et al., 2007) and chemoreceptors (Greenfield et al., 2009). Higher order clustering can facilitate the growth of microdomains by fusion. Indeed, for the synaptic protein Syntaxin, while the small clusters exhibit a gradual decrease in density from the core to the periphery, larger clusters show several density gradients suggesting that these large-clusters are formed by the fusion of several smaller clusters (**Figure 2C**; Bar-On et al., 2012). Tuning the relative distance between microdomains is likely to provide an effective way to modulate biochemical reactions (Lillemeier et al., 2010; Sherman et al., 2011).

## VARIATION IN CLUSTER SIZE DISTRIBUTION

One of the major goals of studies on microdomains is to understand the mechanisms underlying domain formation and regulation. Among the physical observables, cluster size is a key to delineate the different theoretical models, which predict its distribution and how this varies upon changes of parameters such as protein concentration, recycling, or binding rate (Israelachvili, 1985; Turner et al., 2005; Meilhac and Destainville, 2011). Measuring distribution of cluster size is a challenge that PALM microscopy can meet, thanks to molecular counting. Interestingly PALM microscopy has revealed that microdomains can show various types of cluster size distribution. While some membrane proteins (e.g., antiretroviral protein Tetherin, immune protein TCR, G-protein signaling receptor) form cluster with characteristic size of a few to a few 10s of molecules (Lillemeier et al., 2010; Lehmann et al., 2011; Scarselli et al., 2012), others (e.g., Chemotaxis receptor and intercellular adhesion protein) form clusters which follows exponential (Greenfield et al., 2009), or power-law distribution (**Figure 2F**; Truong-Quang et al., 2013). Different types of cluster size distributions reflect distinct mechanisms of formation. An exponential distribution of cluster size, suggests stochastic self-assembly by random receptor diffusion and receptor–receptor interaction (Greenfield et al., 2009), while a power law distribution with exponential cut-off indicates that the size of clusters is regulated not only by dynamical fusion and fission processes but also by endocytosis (Truong-Quang et al., 2013). Analysis of cluster size distributions requires stringent handling as measured distributions can be biased by image processing. For instance, thresholding (consider or eliminate clusters smaller than a certain size) can lead to contradictory conclusions on cluster size (Sherman et al., 2011).**

## CONCLUDING REMARKS

Emerging optical methods have provided a powerful palette to study different aspects of membrane microdomains (**Table 1**). Superresolution optical methods form currently an active field with fast and continuous improvements. In a near future, it is likely that we will gain access to the dynamics of objects with molecular scale precision (2–10 nm) at subsecond time scale. Moreover, combining super-resolution fluorescence microscopy for specificity and localization precision with EM or AFM for resolution will provide molecular details on the organization of supramolecular structures (Watanabe et al., 2011; Chacko et al., 2013). Combination with F-techniques such as FRET, FLIM, and FCS will be also critical to probe the conformation and dynamics of membrane components in microdomains. Thus, questions concerning various aspects of microdomains could be tackled. With a better resolution and multicolor visualization, one could directly visualize the arrangements of molecules within the microdomains. It will be interesting to examine whether molecules are regularly distributed in an array or randomly packed with hollow structure. Quantitative analysis, such as molecular counting, can be very useful to understand the mechanisms underlying domain formation in cell membranes. How the cluster size distribution changes with protein concentration or under perturbations of domain assembly/disassembly rates (e.g., by disruption or enhancement of the actin polymerization) and the endocytosis will help to falsify various theoretical models. Also, by exploring the possibility of the coupling of microdomains between the inner and outer leaflets of the plasma membrane, one could shed light on how information is transmitted through the bilayer. Can clustering of receptors (e.g., GPI-anchored proteins) in the outer leaflet trigger the rearrangement of downstream proteins (e.g., Kinase, phosphatase proteins) in the inner leaflet, thereby amplifying the signals? A similar question could be addressed in the context of cell–cell adhesion for two opposed membranes where cis- and trans-clusters form: are the processes of cis- and trans-clustering occur at the same time? Do cis-clusters pre-exist? Simultaneous visualization of protein clusters at two opposed cell membranes would help solving the above questions. Understanding clustering kinetics is also essential for the understanding on the growth and maintenance of microdomains. Higher temporal resolution will help to probe the dynamics, assembly and disassembly of microdomains in the cell membrane. How passive and active processes driven by trafficking and cytoskeletal interactions integrate to shape clusters is a challenging question, which is now within reach of superresolution methods.

## Conflict of Interest Statement

The authors declare that the research was conducted in the absence of any commercial or financial relationships that could be construed as a potential conflict of interest.
